# Host-microbe interactions in the pathogenesis and clinical course of sarcoidosis

**DOI:** 10.1186/s12929-019-0537-6

**Published:** 2019-06-11

**Authors:** Pleiades T. Inaoka, Masato Shono, Mishio Kamada, J. Luis Espinoza

**Affiliations:** 1 Department of Physical Therapy, School of Health Sciences, Kanazawa University, Kodatsuno, Kanazawa, 577-8502 Japan; 2 Faculty of Medicine, Kindai University, 377-2, Ohno-Higashi, Osaka-Sayama, Osaka, 577-8502 Japan; 3 Department of Hematology and Rheumatology, Kindai University Faculty of Medicine, 377-2, Ohno-Higashi, Osaka-Sayama, Osaka, 577-8502 Japan

**Keywords:** Sarcoidosis, Granulomas, Microbiota, Dysbiosis, Autoimmune disease, Host-microbe interactions

## Abstract

Sarcoidosis is a rare inflammatory disease characterized by the development of granulomas in various organs, especially in the lungs and lymph nodes. Clinics of the disease largely depends on the organ involved and may range from mild symptoms to life threatening manifestations. Over the last two decades, significant advances in the diagnosis, clinical assessment and treatment of sarcoidosis have been achieved, however, the precise etiology of this disease remains unknown. Current evidence suggests that, in genetically predisposed individuals, an excessive immune response to unknown antigen/s is crucial for the development of sarcoidosis. Epidemiological and microbiological studies suggest that, at least in a fraction of patients, microbes or their products may trigger the immune response leading to sarcoid granuloma formation. In this article, we discuss the scientific evidence on the interaction of microbes with immune cells that may be implicated in the immunopathogenesis of sarcoidosis, and highlight recent studies exploring potential implications of human microbiota in the pathogenesis and the clinical course of sarcoidosis.

## Background

Sarcoidosis is a unique inflammatory disease characterized by the formation of non-caseating granulomas that can affect any body organ but most often affects the lungs and lymph nodes and less commonly the skin, eyes, liver, heart, and brain [3, 44]. Extensive research over the last 20 years has contributed to improve the diagnosis and management of this disease. However, despite important advances in the understanding of the inflammatory process associated with sarcoidosis, its etiology still remains unknown. The fact that sarcoidosis can mimic many autoimmune disorders and/or may coexist with them, together with the amelioration of symptoms in response to corticosteroids or immunosuppressive drugs, support the notion that an autoimmune reaction is a critical component on the pathogenesis of this disease [3, 11, 159].

In addition, the existence of cases of familial sarcoidosis along with the observations that certain HLA loci and single nucleotide polymorphisms (SNPs) in non-HLA genes are associated with increased risk of sarcoidosis suggest that a genetic predisposition also plays a role in disease pathogenesis [25, 60]. In addition, accumulating evidence indicate that certain microorganisms, especially *Cutibacterium acne (C. acne)* (previously known as *propionibacterium acne*) and mycobacterium tuberculosis (mTB) may be implicated in the development of sarcoidosis, and thus various authors have proposed the possibility of including antibiotics as part of the standard treatment of this disease [7, 12, 19]. Moreover, given the structural similarities of certain mycobacterial proteins, especially heat shock proteins of mycobacterium tuberculosis (mTB-hsp) with human heat shock proteins (HSPs), it has been proposed that the exposure to mycobacterial antigens, via either natural infection, or by vaccination with BCG, may trigger an autoimmune response leading to sarcoidosis, in genetically prone individuals [36].

With the development of advanced tools for microbe research, such as new generation sequencing (NGS) and the incorporation of more sophisticated methods form microbiome research, it will be possible to determine whether or not, there is a link between specific alterations in the composition of certain microbiota niches (namely dysbiosis of the gut or dysbiosis of the lung microbiota) and the development or progression of sarcoidosis.

In this article, we revise the scientific evidence on the potential role that host/microbe interplay may have in the pathogenesis of sarcoidosis. The clinical relevance of previous reports and ongoing clinical trials testing the therapeutic utility of antimicrobials for the management of this disease is also highlighted.

## Main text

### Epidemiology of sarcoidosis

The incidence rate of sarcoidosis varies depending on the geographic region. For example, high incidence rates are reported in Northern European countries, such as Sweden and Iceland, with nearly 60 cases per 100,000 population [44]. In Asians, the incidence rate is lower, being 1.3 and 2.17 per 100,000 in Japan and Taiwan respectively [131, 159]. In the United States, high incidence in Afro-Americans has been reported (35.5 per 100,000), which contrasts with a lower incidence (10.9 per 100,000) in their Caucasian counterparts [3, 119]. Interestingly, relatively high incidence rates (16.9 per 100,000 per year) have also been documented in African descendants living in certain European countries [45], however, a study examining the occurrence of sarcoidosis in African descendants living in the Caribbeans, reported a much lower incidence (2.3 per 100,000). Although such a lower incidence rate observed in Afro Caribbeans could be due to the most rigorous inclusion criteria utilized in that study, which required a positive biopsy to consider a case to be positive [23], the lower incidence in the Afro Caribbeans may indeed indicate that geographic and environmental factors are also implicated in the pathogenesis of the disease.

Sarcoidosis more commonly occurs in people younger than 40, with a peak in the age-group from 25 to 40 years, but the disease has been diagnosed in people of all ages [3]. Intriguingly, the occurrence of this disease in seasonal clustering has been observed in certain regions of the world. For example, in Japan most cases are diagnosed during June and July. In Spain, half of the cases occur between April and June, and about 70% of cases confirmed in Greece occur between March and May and lower incidence of the disease during the autumn was reported in specific population of the United States [152]. These apparent “seasonal sarcoidosis” could be related to specific seasonal changes in weather, however the seasonal pattern of certain infectious diseases occurrence also points on the possibility that infection with certain microbes may be implicated [57].

Disease severity and clinical manifestations also show certain racial patterns. For instance, in African Americans the disease is more likely to be a chronic and severe disorder involving several organs that can lead to death [102]. Erythema nodosum (an acute, nodular, erythematous eruption that usually is limited to the extensor aspects of the lower legs) occurs frequently in young female Caucasians, especially from Scandinavian countries [44] and Löfgren syndrome (an acute form of sarcoidosis characterized by erythema nodosum, bilateral hilar lymphadenopathy, and polyarthralgia or polyarthritis) is more likely to occur in Scandinavians and in patients from Spain [100]. On the other hand, sarcoidosis with ocular involvement and cardiac features is more likely to occur in Japanese individuals [44].

### Clinics of sarcoidosis

Since sarcoidosis is a multisystem inflammatory disease, symptoms largely depend on the organs affected [12] (Table 1). Strikingly, between 30 to 60% of individuals affected are entirely asymptomatic and the disease is discovered by chance during routine medical checkups. Symptoms, when occur, tend to be non-specific and may include fatigue, anorexia, lack of energy and arthralgia that may mimic a variety of conditions such as malignancies, autoimmune disorders or chronic infections [16, 20, 100].

**Table 1:** Demographic characteristics and main clinical features of Sarcoidosis

Demographic data	Main characteristics	Reference
Gender	No predominance	
Age at onset (years)	Any age Peaks at 25~40 years. Nearly 30% of cases in older than 60	[3]
Ethnicity	More common in northern Europeans (60 per 100,000). Less common in Asians (1.3–2.17 per 100,000) In the USA (35.5 per 100,000 in Afro-Americans 10.9 per 100,000 in Caucasians)	([3]; [15])
Clinical findings		
Constitutional manifestations	Asymptomatic (30~60% of cases) Malaise, Fever, Anorexia, weight loss	([15]; [86]; [102]; [109])
Pulmonary Sarcoidosis	At least 90% of affected individuals have lung involvement -Most patients are asymptomatic. -Primarily manifests as hilar or mediastinal adenopathy -Some patients present with interstitial lung disease -Fibrosis of the lung (20% of patients) -Pulmonary hypertension (in 5% of cases)	([15]; [84]; [102])
Skin sarcoidosis	Skin lesions (found in 20~35% of patients) and can cause: Rash, papules or Erythema nodosum	([44]; [67])
Löfgren syndrome	Occurs more often in Scandinavian. Fever, Enlarged lymph nodes, Arthritis and erythema nodosum	[87, 100]
Ocular sarcoidosis	Can affect any part of the eye and may cause: Uveitis, Scleritis, Conjunctival-granuloma, Eyelid abnormalities, Optic neuropathy, lacrimal gland enlargement and orbital inflammation	([70]; [115])
Musculoskeletal	Infrequent May cause: Nonspecific arthralgia, Polyarthritis (acute or chronic) Sarcoid myopathy (muscle weakness, muscle pain, or muscle nodules)	[109]
Cardiac sarcoidosis	Infrequent. Most commonly manifests with arrhythmias (tachycardia or heart block). Less commonly: Pericardial effusion or myocardial granulomas leading to cardiac fibrosis	([63]; [86]; [132])
Neurosarcoidosis	Not well characterized due to its rarity. Patients may present with: Intracranial or spinal mass lesions Optic neuritis Facial mononeuropathies Myopathy and peripheral neuropathy	[79]

Pulmonary involvement, which is the most frequent form of the disease, may present with cough and shortness of breath and less commonly hemoptysis [15, 84]. The second most commonly affected organ is the skin and may present with variable rash, papules or with the typical bump nodules of erythema nodosum [9, 67]. In individuals with eye involvement, symptoms may include dry eyes, blurry vision and red eye [67].

Except for Japanese individuals, in whom cardiac sarcoidosis is a common manifestation of this disease, heart affectation has been considered a rare phenomenon [73]. However, recent publications suggest that cardiac sarcoidosis is more common than previously reported with most patients unaware of its presence [86]. Clinically, cardiac sarcoidosis may present with severe dysrhythmias, such as tachycardia or heart block, both of which can be fatal [63, 161]. Less commonly, patients with cardiac sarcoidosis present pericardial effusion or other structural lesions such as granuloma formation and fibrosis, leading to mechanical dysfunction of the heart [63].

Furthermore, sarcoidosis can cause a wide variety of musculoskeletal complaints affecting the bones, joints, or muscles with joint involvement manifested as acute or chronic arthritis [109, 137]. Curiously, in Japanese individuals, enlargement of spleen has also reported in a considerable number of patients with sarcoidosis, which was linked to a specific genetic background [129].

Finally, Sarcoidosis affecting the nervous system (neuro-sarcoidosis) is a rare condition that can manifest as a space-occupying lesion in the central nervous system or as peripheral neuropathies [79, 125, 162].

Findings in conventional Chest X-rays may be the first clue that suggest the diagnosis of sarcoidosis, which is complemented with more advanced imaging techniques such as high resolution CT scan (HRCT) and magnetic resonance (MRI) imaging.

In addition, 18F-fluorodeoxyglucose positron emission tomography–computed tomography (FDG-PET/CT) scan has become an extremely useful technique not only for the diagnosis of sarcoidosis but also for evaluating treatment response [62, 84, 100], however the high cost of FDG-PET/CT scan is an important limitation for its utilization as a standard approach for monitoring patient response to therapy.

In patients with lung involvement, pulmonary function tests are useful to assess lung functioning and bronchoscopy for bronchial inspection and biopsy extraction are frequently utilized. The development of ultrasound imaging coupled with bronchoscopy has improved the diagnosis of pulmonary sarcoidosis as it increases the yield of tissue aspiration of hilar and/or mediastinal lymph nodes [84].

According to the inclusion criteria from the American Thoracic Society/European Respiratory Society/World Association of Sarcoidosis and other Granulomatous Disorders (ATS/ERS/WASOG), there are three criteria for diagnosis sarcoidosis: (1) a compatible clinical and radiologic presentation, (2) pathologic evidence of typical lesions in more than one organ, and (3) exclusion of other diseases known to cause granulomatous disease. However, since the clinical presentation of sarcoidosis is quite variable and there is not a single diagnostic test or procedure to definitely diagnosis sarcoidosis, in many cases the diagnosis of this disease is challenging [74]. Recently, Bickett et al. reported the development of quantitative diagnostic criteria for sarcoidosis. This approach combines biopsy findings in granulomas (SDS biopsy score) and clinical features (SDS clinical score). Although this study included patients from a single center, the SDS score performed well (specificity of > 95%) as a diagnostic test for sarcoidosis [14], and thus this score may be a “first step in making the diagnosis of sarcoidosis significantly less arbitrary”[84]. Further analyses, ideally conducted across several institutions are required to determine the diagnostic utility of this score.

A recently study that included 2163 Caucasian patients with sarcoidosis who were evaluated at 31 study centers in Europe, revealed that patients with acute onset were mainly female, young and presented with scadding type I or II. High frequency of eye and skin involvement along with fatigue were also frequently observed in female patients. More importantly, according to the predominant organs affected, patients could be consistently stratified into five distinct subgroups: 1) abdominal organ involvement, 2) ocular–cardiac–cutaneous–central nervous system disease involvement, 3) musculoskeletal–cutaneous involvement, 4) pulmonary and intrathoracic lymph node involvement, and 5) extrapulmonary involvement [135]. In addition to its utility for stratifying homogenous and well-defined subgroups of sarcoidosis patients, these findings are very useful to direct new studies aimed to identify potential links between specific genotypes with disease phenotypes. This is particularly important considering that a genetic predisposition appears to play important roles in the development and clinical course of sarcoidosis.

Treatment recommendations for patients with sarcoidosis will not be reviewed in this article, however, it is important to mention that since spontaneous resolution frequently occurs, many patients with pulmonary disease do not require any specific treatment and can be monitored over a period of time [10]. In those requiring treatment, oral corticosteroids, such as prednisone or prednisolone, remain the first line therapy in both, acute and chronic sarcoidosis and in most cases the disease is very responsive, however, there are no standard protocols for corticosteroid dose or duration of treatment and long-term exposure to these agents is associated with substantial morbidity [83].

In patients who do not respond to corticosteroids, more potent immunosuppressant drugs such as methotrexate, azathioprine and mycophenolate may be required, although due to the considerable secondary effects associated with the long-term use of these drugs, patients need to be carefully monitored [10, 126].

In addition, biological agents, including anti-TNF-α monoclonal antibodies (infliximab) and anti-CD20 monoclonal antibodies (rituximab) have shown promising therapeutic potential in select group of patients, especially in those with severe or refractory sarcoidosis, [1, 83, 126].

Recently the Innate Repair Receptor (IRR) activator ARA 290, has been designed an orphan drug for the treatment of sarcoidosis by the U.S. Food and Drug Administration (FDA) and the European Union and was granted as Fast Track designation by the FDA for the treatment of painful small fiber neuropathy in patients with sarcoidosis. ARA290 has been evaluated in patients with small fiber neuropathy associated with sarcoidosis or type 2 diabetes mellitus. In the sarcoidosis group, ARA290 improved quality of life and significantly ameliorated neuropathic and autonomic symptoms and thus ARA290 will be useful for ameliorating pain associated with this form of sarcoidosis [28, 155].

### Pathogenesis of sarcoidosis: the autoimmune theory

As mentioned above, the key pathogenic component of sarcoidosis is the development of noncaseating granulomas that can arise in different organs. Current evidence suggests that a combination of genetic predisposition and environmental conditions play a central role in the excessive immune reaction leading to the development of sarcoidosis [54, 125]. Granulomas constitute an excessive immune response aimed to control or eliminate an uncharacterized antigen. Proposed candidate antigens include various microorganisms or their products, inorganic particulate matters, metal particles or unknown environmental contaminants and due to the predominance of lung involvement, antigens are believed to enter the body via the respiratory system within airdrops or microparticles [35, 91].

Histologically, sarcoidal granulomas are characterized by the presence of centrally organized collections of epithelioid histiocytes and macrophages surrounded by giant cells and lymphocytes, mostly Th1 lymphocytes, and a rim of fibrosis [21, 91]. Unlike infectious granulomas, such as those associated with mTB, in sarcoidal granulomas, necrosis is uncommonly seen, which defines its non-caseating nature [21, 147]. Although not specific to sarcoidosis, Schaumann bodies (within the cytoplasm of multinucleated cells) and asteroid bodies (star shaped cytoplasmic inclusions) are frequently seen [122].

**Fig. 1:**
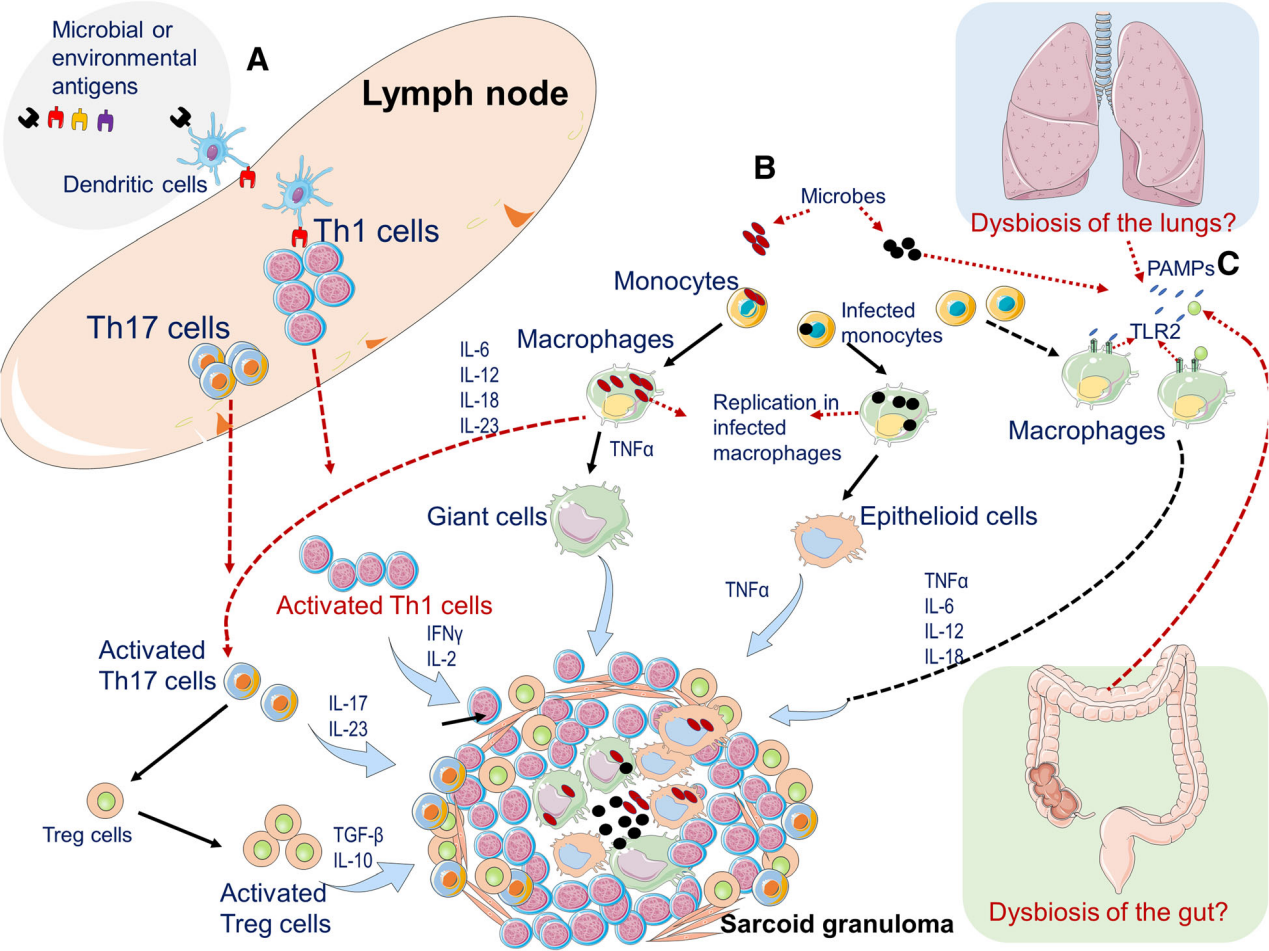
Legend of the figure. A schematic model for granuloma formation. **a** Dendritic cells pick up the initiating antigen (likely an environmental airborne antigen or a microbe) and migrate toward lymph nodes, where they interact with appropriate T cells, promoting the differentiation and clonal expansion of T helper (Th)1 and 17 cells. Activated Th1 and Th17 cells promote an inflammatory response via the release of cytokines such as interferon γ (INF-γ), interleukin 2 (IL-2) and IL-17 contributing to granuloma formation. **b** Microbes, such as mycobacteria or *C. acnes*, may directly infect monocytes or macrophages, which fail to properly eliminate the infection and in turn differentiate into giant cells or epithelioid cells. Simultaneously, giant cells or epithelioid cells produce tumor necrosis factor-α (TNF-α), which promotes the formation and maintenance of sarcoid granulomas. In addition, by secreting MCP-1 and CXCL10 chemokines, macrophages attract Th1/17 cells, monocytes, regulatory T cells (Tregs). These Tregs fail to control the inflammatory response and secrete transforming grow factor β (TGF- β) that may contribute to fibrosis and granuloma organization (**c**) An altered composition of the microbiota (dysbiosis) of the gut or lungs may act as a source of microbes that secrete pathogen-associated molecular patterns (PAMPs), which may activate immune cells of the innate system, such as monocytes or eosinophils, by interacting with pathogen recognition receptors (PRRs) like toll-like receptors (TLRs) and in the presence of sustained activation, it may further promote the generation of giant cells and epithelioid cells from macrophages and thereby contribute to granuloma formation via the secretion of cytokines such as IL-6, IL-12, IL-18, and TNF-α

The immune signature of sarcoidosis is the excessive immune response mediated by CD4+ type 1 helper-like cells (Th1-cells), including hyperactivation of Th1-cells and increased levels of Th1 cytokines (Fig. 1). Based on studies using bronchoalveolar lavage fluids (BALF) samples, it has been documented that in patients with pulmonary sarcoidosis, the local microenvironment is characterized by a Th1/Th2 imbalance in which Th1-related cytokines, such as interleukin-2 (IL-2), IL-12, interferon-γ (IFN-γ), and tumor necrosis factor α (TNF-α) promote a persistent inflammatory response in the affected tissues [29]. For example, both IL-2 and IFN-γ are potent inducer of T cell proliferation and TNF-α promotes the differentiation of macrophages into giant cells and contributes to granuloma formation [164].

Another subset of CD4T-lymphocytes, involvement in granuloma induction or maintenance in sarcoidosis are Th17 cells, as documented by the elevated numbers of IL-17, IL-22 and IFN-γ secreting CD4T-lymphocytes found in the blood of patients with sarcoidosis, as well as an increased proportion of Th17 cells, located in and around sarcoidal granulomas [127, 144, 151]. These findings indicate that Th17 cells are pathogenic in sarcoidosis and suggest that the inhibition of both Th1/Th17 pathways may be required to achieve therapeutic efficacy in sarcoidosis patients [66].

Regulatory cells (Treg cells), which are defined as CD4^+^CD25^bright^FoxP3^+^ have been also implicated in the pathogenesis of sarcoidosis. Treg cells are a subset of lymphocytes that inhibit autoimmune reactions by controlling the proliferation of CD4^+^ and CD8^+^ T lymphocytes via secretion of immunosuppressive cytokines such as interleukin 10 (IL-10) and transforming growth factor β (TGF-β) or through mechanisms dependent on cell contact via the CD25 molecules constitutively expressed on their surfaces [127]. High number of Treg cells are frequently found in lymph nodes and in BALF of patients with sarcoidosis and accumulate at the periphery of sarcoid granulomas. Notably, although these cells exhibit powerful antiproliferative activity, they fail to completely inhibit TNF-α production [29] likely due their increased apoptotic susceptibility [18]. Contrary to the local dysfunction of granuloma associated Treg cells, increased circulating Treg cells have been documented in the blood of patients with sarcoidosis, which may explain the apparent immune anergy frequently observed in these patients. Experimental studies have shown that Treg depletion accelerates in vitro granuloma growth in mononuclear cell cultures of healthy controls, but not in those from patients with active sarcoidosis indicating that, although healthy Tregs suppress the initial steps of granuloma formation, they have no positive influence on sarcoidosis lesions [142]. Notably, a significant increase in the Th17/Treg cell ratio has been reported in sarcoidosis patients [78], which is consistent with other studies reporting that in patients with pulmonary sarcoidosis in relapse after corticosteroid withdrawal, a significant increase in circulating Th17 cell along with a concomitant decrease in Treg cell has been documented [95]. Interestingly, a recent study that analyzed samples from airways obtained by BAF, Tregs from the lung of sarcoidosis patients were characterized by expressing high levels of the receptor CD278 on their surface and such a CD278 expressing Tregs cells were restricted to the inflamed lungs and were absent in blood Tregs of sarcoidosis patients as well as in lung and blood Tregs of healthy volunteers. Strikingly, CD278 expression was particularly high on Tregs derived from the lungs of Löfgren’s syndrome patients who present with acute disease and that often resolves spontaneously [128] indicating that CD278 may be a marker of disease activity and could be considered a biomarker for the prognosis of sarcoidosis.

### Pathogenesis of sarcoidosis: genetics and environmental factors

The appearance of familiarly clustered cases of sarcoidosis and the racial differences in disease incidence and clinical progression support a possible link of genetics with the development of this disease [104, 116, 154]. These observations are substantiated by the findings of a large study recently conducted in Sweden that included 23 880 individuals with sarcoidosis assessed between1964 to 2013 and matched (10:1) to general population controls. A heritable link was tracked in 39% of cases (95% CI 12–65) analyzed and having ≥1 first degree relative with sarcoidosis was associated with a 3.7-fold increase in the risk for sarcoidosis (95% CI 3.4–4.1) and the risk increased in those with ≥2 relatives (RR 4.7) and in Löfgren’s syndrome (RR 4.1) [123].

The most strongly and consistently associated risk factor for sarcoidosis is the major histocompatibility complex (MHC) region, comprising of HLA class I (HLA-A, −B, and -C) and class II (HLA-DP, −DQ, and -DR) genes, being HLA class II variants, particularly HLA-DRB1 and DQB1 the alleles most prevalently reported in association with sarcoidosis [60, 158]. For example, B1*0301/DQB1*0201 is strongly linked with Löfgren’s syndrome and it predicts good prognosis in various populations [71]. A decreased risk of sarcoidosis has been reported in individuals carrying the HLA class II allele *HLA-DRB1*^*∗*^*01/*^*∗*^*04* (Fingerlin, Hamzeh and Maier, 2015; [104, 130]). Individuals with the allele *HLA-DRB1*^*∗*^*14:01* more likely develop a chronic disease [60, 61, 123], and those with HLA-DRB1*03:01-DRB3*01:01are more likely to have pulmonary sarcoidosis [90, 158].

On the other hand, SNPs in non-HLA genes, including *CCR2, CCR5, IL1A, IL23R, TNF-α* and *NOD2, FCGR,* have been also reported in association with the disease [61, 121, 139]. It must be noted, however, that many of these gene-association studies were conducted in single populations where allele frequencies in sarcoidosis patients were compared with healthy control individuals and most of those studies did not include a confirmatory cohort. Not surprisingly, only a few of the reported gene associations have been consistently validated across distinct populations (Fingerlin, Hamzeh and Maier, 2015). Interestingly, genetic variants in TNFα gene, as a susceptibility factor for sarcoidosis, have been replicated across populations in various studies (Fingerlin, Hamzeh and Maier, 2015; [104]) and some of them have been also verified in meta-analyses [59, 138].

Despite that numerous genetic risk factors in association with sarcoidosis have been identified, in the majority of cases, the biologic functions of specific variants and their precise interaction with environmental exposures remain unknown. The use of new generation sequencing based HLA typing will be helpful to identify potential new associations and to elucidate the specific contribution of particular gene variants, especially within the HLA region, to disease susceptibility and may provide new insights on the pathogenesis of this disease.

Sarcoidosis has been also linked with certain environments factors such as exposure to inorganic particles, insecticides, and moldy environments. Occupational studies have also found an increased risk of sarcoidosis among metalworkers and fire fighters [110]. In a recent epidemiological study, significantly elevated rates of this disease were observed among workers that responded to the world trade center terror attack in 2001. The increased risk was observed among workers at all exposure levels [157], suggesting that nanoparticles or metal and chemicals fumes likely derived from the occupational domain or other environmental contaminant may act as important triggers of this disease.

Furthermore, tattoos-induced sarcoidosis has been described in the literature, and although the etiology of this phenomenon is unknown, it has been attributed to an excessive immune reaction against pigment particles, in genetically predisposed individuals. Notably, the sarcoidal granulomas, contain pigment particles, pigment laden macrophages and polarizing material and some patients may develop symptoms of pulmonary sarcoidosis [118].

In line with these observations, it has been proposed that sarcoid formation may occur in the context of infectious and non-infectious causes, depending on the genetic background of the host. In this model, the exposure to certain microorganisms expressing microbial hsps or pathogen-associated molecular patterns (PAMPs), which are sensed by immune cells via pattern recognition receptors (PRR), may generate an increased and persistent release of both human HSPs and microbial hsp. Given the high homology of mycobacterial hsp, particularly *mycobacterium tuberculosis* hsp (mTB-hsp) and HSP, this may lead to an excessive immune response that ultimately may cause sarcoidosis. On the other hand, in non-infectious causes of sarcoidosis, the persistent exposure to an array of damaging factors such as phagocyted metal fumes, pigments and other chemicals may result in the release of damage-associated molecular patterns (DAMPs), also known as danger-associated molecular patterns, which are host biomolecules capable of initiating and perpetuating a noninfectious inflammatory response [37].

Intriguingly, patients with sarcoidosis have increased risk of developing lymphomas, a condition that has been called sarcoidosis-lymphoma syndrome, with sarcoidosis preceding lymphoma in most cases, though sarcoidosis following lymphoma has been also reported [96] and sarcoidosis or sarcoid-like granulomas are being frequently reported in patients with cancer, particularly in patients receiving immunotherapy with immune checkpoint inhibitors [32, 55, 69, 146], which has been attributed to an excessive immune response against cancer cells, which may be activated by immunotherapy agents and thus substantiating the critical role that an excessive immune response plays in the pathogenesis of sarcoidosis. Experimental data indicate that the immune check point blockade augments the number of circulating Th17 CD4+ cells along with an increased production of proinflammatory molecules, such as IL-6 and TNF-α [69, 156], which may promote the formation of granulomatous/sarcoid-like lesions. Interestingly, a recent study showed that PD-1 pathway is upregulated in sarcoidosis. In this study, sarcoidal PD-1 + CD4 + T cells displayed reduced proliferation rate, their proliferation capacity could be recovered after treatment with anti-PD-1[17]. These findings suggest a potential benefit and a dual role of PD-1 blockade in sarcoidosis in an analogous way as TNF-α blockers.

### Microbes and sarcoidosis

The potential involvement of microbes in the pathogenesis of sarcoidosis was proposed many years ago [35, 49]. This was mainly based on the observations that granulomas observed in sarcoidosis share certain histological similarities with the granulomatous lesions caused by pathogen infections such as leprosy, tuberculosis and some parasitic infections [2, 148]. To identify potential pathogens linked with sarcoidosis, a variety of sarcoidosis-specimens have been analyzed utilizing several techniques, including microbe culture, real time PCR, immunohistochemistry and proteomic and genomic studies [25, 47]. As a result, various microorganisms have been detected in sarcoid granulomas and other tissues affected by sarcoidosis, which suggests that microbes may have a role in the pathogenesis of this disease [22, 25, 35, 97]. However, it is important to notice that most of the putative microorganisms have been identified at genomic levels (DNA or RNA) and only a few studies have documented the presence of microbes using bacteriological or proteomic methods (Table 2). The considerable variation among different reports and the fact that the presumptive microbes are not detectable in all sarcoidal granulomas or in all patients analyzed, along with the detection of those microorganisms, even in tissues of normal individuals or in samples derived from patients with other diseases, constitute important limitations of the microbial theory and therefore, current evidence does not support a direct causative role of microbes in sarcoidosis.

**Table 2:** ^a^ List of putative microbes associated with sarcoidosis and detected by microbiological or proteomic methods

Microorganism	Detection method	Main findings	Type of sample	Reference
*C. acnes* and Cutibacterium sp.	IHC with a *P*. *acnes*-specific monoclonal antibody.	C. *acnes*-positive reactivity in 10 (63%), of granulomas form cardiac sarcoidosis	FFPE of myocardial tissues obtained by surgery or autopsy and endomyocardial biopsy from patients with cardiac sarcoidosis (*n* = 26), myocarditis (*n* = 15), or other cardiomyopathies (*n* = 39)	[5]
	IHC using monoclonal antibody against *P. acnes*.	Granuloma in the epiretinal membrane was observed in 4 of 10 patients with sarcoidosis, and all the granulomas were positive for PAB	Ten patients with uveitis associated with sarcoidosis	[70]
	IHC with a *P. acnes*-specific monoclonal antibody (PAB antibody).	*P. acnes*, identified as round bodies that reacted with the PAB antibody, were present in 10/12 samples (83%) from 9/11 patients (82%) with sarcoidosis	Eleven patients (12 eyes) with sarcoid uveitis were enrolled in this study.	[107]
	MALDI-IMS for propionibacterial proteins	Sarcoidosis 7/15 Controls 1/4	Nineteen snap frozen sarcoidosis specimens were analyzed using a MALD-IMS	[113]
	IHC and western blotting with PAB antibody and ribosome-bound trigger-factor protein (TIG antibody)	Small round bodies within sarcoid granulomas in 20/27 (74%) video-assisted thoracic surgery lung samples, 24/50 (48%) transbronchial lung biopsy samples, 71/81 (88%) Japanese lymph node samples, and 34/38 (89%) German lymph node samples.	FFPE samples of lungs and lymph nodes from 196 patients with sarcoidosis, and corresponding control samples from 275 patients with non-sarcoidosis diseases.	[108]
	Isolation of *P. acnes* in culture	Sarcoidosis 31/40 Controls 38/180		Abe, 1984 [19]
mTB and Mycobacterium sp	IHC with panels of various antibodies, including antimycobacterial MAbs specific for M tuberculosis complex, for M. leprae and for cross-reactive mycobacterial antigens	Pleomorphic chromogens (PCs) structures are sites of mycobacterial degradation	28 cases of sarcoidosis and 14 cases of malignancy associated sinus histiocytosis (series B)	
	Microscopic identification of mTB bacilli	Tubercle bacilli found in the sarcoid phase in 18; in preceding caseating tuberculosis in 11;.	Granuloma specimens from 240 patients with tuberculosis.	[133]
	IHC using mTB PPD antibody was used to detect mycobacterial antigens	IHC analysis for MTB anti-PPD antibody positive for all TB patients and in 3 sarcoidosis patients (30%)	FFPE tissue specimens from granulomatous tissues of 10 patients with sarcoidosis and 12 confirmed pulmonary tuberculosis	
Borrelia sp and Borrelia Burgdorferi	IHC with anti-borrelia polyclonal antibody and assessment with FFM	Using FFPE, Borrelia were detected in 127 cases of GA (80.9%).	157 biopsies of GA	Ziemer M, 2008 [165], Germany
	IHC with anti-borrelia polyclonal antibody and assessment FFM	26% (13/35) were positive to Borrelia sp. 1/61 Borrelia sp.	Cutaneous sarcoidosis (skin biopsies)	Derler, 2009 [30] Austria
	Dot-blot analysis (Dotblot Borrelia Kit) and ELISA assay	15/46 Borrelia sp. 2/100 Borrelia sp.	Serum samples from 46 patients with sarcoidosis an 150 controls	Ishihara, 1998 [82] Japan
	ELISA and Western blot for Borrelia burgdorferi	B. burgdorferi + in sarcoidosis 1/60 controls 27/1000 Borrelia burdorferi.	Serum samples os patients with sarcoidosis	Martens, 1997 [98] Germany
	Elisa and Dot-blot analysis for Borrelia sp.	Sarcoidosis patients 2/38 (5.3%) Borrelia sp. Control 1/80 (1.2%) Borrelia sp.	Serum samples Mainly lung sarcoidosis	Ishihara, 1996 [81] Japan

^a^Selected references are articles in which microbes or their proteins were directly documented within sarcoidal granulomas. Studies where the presence of microbes was investigated at genomic level (DNA or RNA) are compiled in other reviews articles published elsewhere

Immunohistochemistry (IHC)

Matrix-assisted laser desorption ionization imaging mass spectrometry (MALDI-IMS)

Focus-floating microscopy (FFM)

Purified protein derivative (PPD)

Formalin-fixed paraffin-embedded (FFPE)

Granuloma Annulare (GA)

In addition, a putative involvement of microbes in the pathogenesis of sarcoidosis is indirectly supported by studies reporting considerable activation PRRs, which are essential components of the innate immune system involved in the control of infective microorganisms. Among the altered PRRs, enhanced expression toll-like receptors (TLR) and their activated downstream signals have been documented in various cell subsets of patients with sarcoidosis. For example, a study that assessed global transcriptomic changes of miRNAs of circulating Tregs and CD4+/CD25 + Tregs from BAL, identified alterations in TLR-2 signaling pathway. Notably, induction of TLR-2 expression was found not only in Tregs, but also in the heterogeneous population of peripheral blood mononuclear cells of patients with pulmonary sarcoidosis [85]. In addition, increased inflammatory cytokine secretion was reported when immune cells obtained from BAL of sarcoidosis patients were in vitro exposed to LpqH, a 19kd lipoprotein of mTB, that is a ligand of TLR2[64]. In the same study, TLR-2 gene deletion in a murine model of Th1-associated lung disease induced by heat-killed *C. acnes,* resulted in a considerable attenuation of the granulomatous pulmonary inflammation compared to wild-type C57BL/6 animals [64]. Another study found a significant association between TLR9 expression and CD4^+^ lymphocytes in BAL of patients with sarcoidosis. Increased TLR9 expression in alveolar macrophages derived from patients with sarcoidosis was also reported and these cells secreted higher levels of cytokines in response to in vitro stimulation [134]. TLR9 is an important receptor mainly expressed by dendritic cells, macrophages and NK cells that recognizes specific unmethylated CpG motifs of DNA present in bacteria and viruses. This receptor triggers signaling cascades leading to a pro-inflammatory cytokine response has attracted considerable attention for its immunotherapeutic potential [58].

So far, the most comprehensive study aimed to clarify the role of microbes in sarcoidosis is a meta-analysis that included 58 case–control studies (involving more than 6000 patients in several countries) reporting the presence of microorganisms in samples of patients with sarcoidosis using culture methods or molecular biology techniques concluded that an etiological link exists between *C. acnes* and sarcoidosis (OR 18.80, 95% CI 12.62, 28.01). Strong association was also found with mycobacterium tuberculosis (mTB), as this bacterium was detected within the sarcoidal lesions of nearly 25% of sarcoidosis patients (OR 6.8). Other microbes, including *Borrelia*, HHV-8, as well as *Rickettsia helvetica*, *Chlamydia pneumoniae*, Epstein-Barr virus and Retrovirus were not associated with sarcoidosis [54]. In the next section, we will discuss additional studies supporting a causal association between *C. acnes* and mTB with sarcoidosis.

### *Role of C. acnes* and sarcoidosis

*C. acnes,* is a slow-growing anaerobic Gram-positive bacterium rod that is etiologically linked to the skin disorder acne. In addition, *C. acnes* can cause chronic blepharitis, and endophthalmitis and is an opportunistic agent that can cause endocarditis, corneal ulcer, septic arthritis and is a contaminating agent of areas subcutaneous such as after craniotomy. This bacterium is largely commensal and part of the skin microbiota detectable on healthiest adult humans’ skin and may be also found throughout the gastrointestinal tract.

*C. acnes* can be found in BAL of the majority of patients with sarcoidosis and its presence has been associated with disease activity, however the microbe can be also found in around 20% of controls [46]. In addition, *C. acnes* has been identified in granulomas and inflammatory cells of myocardial tissues [5], lymph nodes [141] neurological system [160], the granulomas present in the epiretinal membrane excised from patients with uveitis associated with sarcoidosis [70] and many other sarcoidal granuloma specimens. Importantly, *C. acnes* constitutes the only microorganisms that has been isolated from sarcoidal specimens by bacterial culture [47].

One of the first attempts to systematically study a potential link between sarcoidosis and pathogens was an international study included formalin-fixed and paraffin-embedded (FFPE) sections of lymph nodes of patients from Japan, Italy, Germany, and England. The specimens (108 sarcoidosis, 65 tuberculosis and 86 control samples) were subjected to quantitative real-time PCR to identify genomes of *C. acnes*, Propionibacterium granulosum, mTB, Mycobacterium avium subsp. paratuberculosis, and Escherichia coli (as the control). The authors concluded that Propionibacterium spp. are more likely than Mycobacteria spp. to be involved in the etiology of sarcoidosis and the association occurred not only in Japanese but also in European patients with sarcoidosis [48]. High frequency of *C. acnes* genomes were also found in FFPE derived from Chinese patients with sarcoidosis but not in tuberculosis or lung cancer [163].

Negi and colleagues examined formalin-fixed and paraffin-embedded samples of lungs and lymph nodes from 196 patients with sarcoidosis, as well as control samples derived from 275 patients with non-sarcoidosis diseases. Using immunohistochemistry with *C. acnes*-specific monoclonal antibodies that react with cell-membrane-bound lipoteichoic acid (PAB antibody), the authors detected small round bodies within sarcoid granulomas in 20/27 (74%) video-assisted thoracic surgery lung samples, 24/50 (48%) transbronchial lung biopsy samples, 71/81 (88%) Japanese lymph node samples, and 34/38 (89%) German lymph node samples. Notably, PAB antibody did not react with non-sarcoid granulomas in any of the 45 tuberculosis samples or the 34 samples with sarcoid reaction, suggesting that this indigenous bacterium might be the cause of granuloma formation in many sarcoid patients [108].

*C. acnes* secretes propionic acid and other short-chain fatty acids metabolites (SCFAs) such as formic acid (C1), acetic acid (C2), butyric acid (C4), and valeric acid (C5). Interesting SCFAs secreted by gut microbiota exert modulatory effects on the immune system, including the differentiation and proliferation of Treg cells [4, 89], and regulate the production of blood cells in the bone marrow in the mouse [88, 150].

One may expect that propionic acid released by *C. acnes* may promote the proliferation of Treg cells and thus it could theoretically prevent the development of sarcoidosis rather that promoting granuloma formation, however, in individuals with new onset pulmonary sarcoidosis, a large infiltration of Treg cells, along with Treg cytokines such as IL-10 and TGF-β are increased but are not functional [29]. Therefore, it is plausible that Treg induction via excessive production of propionic acid in the site of inflammation may promote granuloma formation in sarcoidosis, however more controlled studies, using more individuals and testing tissues from other organs affected by sarcoidosis are needed to clarify these issues.

In summary, there is abundant laboratory and clinical evidence supporting an association between sarcoidosis and *C. acnes*, however is worthy to notice that the vast majority of the studies reported so far have been conducted by Japanese groups testing Japanese patients, while only a few testing specimens obtained from patients from African American or Caucasian patients having been published. More studies are needed to demonstrate a direct causal link between this pathogen and sarcoidosis.

### *Role of mTB* in sarcoidosis

The link between mTB and sarcoidosis was proposed many years ago [76] given the fact that sometimes distinguishing between sarcoidosis and tuberculosis in the clinical setting can be challenging, especially in tuberculosis cases with atypical presentation or in areas where tuberculosis is endemic [105]. The differential diagnosis sometimes is difficult because both diseases mainly affect the lungs and frequently involve the enlargement of mediastinal, perihilar and other thoracic lymph nodes with marked radiological similarities [101]. In addition, both diseases may present with similar extrapulmonary manifestations in the skin, kidneys and musculoskeletal system and may cause systemic symptoms such as fever, malaise, and weight loss [94]. Moreover, the presence of granulomas is a histopathological features shared by both diseases and, although tuberculosis typically causes caseating granulomas, some patients may present with non-caseating granulomas [8]. Furthermore, as mentioned before, current standard treatment of sarcoidosis involves the administration of corticosteroids whose prolonged use results in immunosuppression. As a result, latent tuberculosis reactivation is relatively frequent in sarcoidosis patients taking corticosteroids adding an additional layer of complexity to the distinction between the two diseases [94, 103].

Pathological studies have documented the presence of mTB-hsps, including mTB-hsp70, mTB-hsp65, and mTB-hsp16 in lymph node tissues of patients with sarcoidosis, being mTB-hsp16 predominantly detected in the early stage of the disease and mTB-hsp70 in stage II sarcoidosis [38]. As expected, mTB-hsps are also detectable in tuberculosis, however higher nitrate/nitrite concentrations are observed tuberculosis than in sarcoidosis. Since nitrite produced by mTB in infected macrophages modulates mycobacterial survival [27], it has been proposed that lower levels of nitrate/nitrite may induce a “mTB genetic dormancy program” via higher mTB-hsp16 expression favorable for the development of sarcoidosis [39]. Given the structural similarities between mTB-hsps and human-HSP, it has been proposed that the exposure to mycobacterial antigens via either natural infection, or by vaccination with BCG, may induce an autoimmune response in genetically prone individuals that may result in sarcoidosis [36]. In line with these observations, in patients with sarcoidosis, specific antibodies to these mycobacterial antigens (anti- mTB-hsps) are frequently detected in serum samples. Interestingly, in comparison with tuberculosis, significantly higher levels of anti-mTB-hsp70 antibodies are observed in sarcoidosis patients [40].

There is a relative high prevalence of sarcoidosis in certain countries of northern Europe and for many years it was assumed that whereas sarcoidosis was a disease of the developed world, tuberculosis was a disease that mainly affect people in the developing world [6]. However, with the introduction of new diagnostic modalities such as high resolution computed tomography, and transbronchial lymph node and lung biopsies, sarcoidosis cases in developing countries are being increasingly detected and thus recent reports suggest that sarcoidosis is a worldwide disease.

Early observations published during the mid-twentieth reported the presence of mTB bacilli-like structures identified by bacteriological methods in inflamed tissues of some patients with suspected sarcoidosis [26, 120, 133], although those findings were not consistently observed in all the specimens examined and some of the patients studied may have indeed presented both diseases concomitantly (tuberculosis and sarcoidosis), given the higher prevalence of tuberculosis in that period and the fact that immunological anergy associated with sarcoidosis is a risk factor for the development of tuberculosis. With the introduction of the polymerase chain reaction (PCR) analysis in medical research at the beginning of the 1990s, several investigators independently reported the finding that mycobacterial DNA can be frequently detected in tissues specimens from patients with sarcoidosis [68, 124] and the causal link of this mTB and other mycobacteria with sarcoidosis was further tested in the following decade using more advanced microbiological and molecular techniques [112, 145].

A meta-analysis assessing the molecular evidence of the role of mycobacteria in sarcoidosis analyzed 31 studies in which PCR for nucleic acid amplification followed by identification of nucleic acid sequences specific for different types of mycobacteria concluded that 231 out of 874 patients were positive for mycobacteria with a positive signal rate of 26.4 (23.6–29.5%), and the odds of finding mycobacteria in samples of patients with sarcoidosis versus controls were 9.67 (4.56–20.5%). It must be noted however that methodological and statistical heterogeneity and publication bias was evident in most of the studies assessed [72]. However, similar results were reported by another meta-analysis that assessed 36 different studies (1034 sarcoidosis patients and 1054 control), where the positive signal rate was 24.2% (250 sarcoidosis samples were positive for some form of mycobacteria DNA) [54].

A comprehensive assessment of immune infiltration of sarcoidosis or tuberculosis granulomas using immunohistochemistry revealed that, although there was no significance difference in single CD4+, CD8+, CD22+, CD14+ and CD68+ staining between sarcoidosis and tuberculosis sections, CD163 expression (a marker of M2 macrophages) was significantly increased in sarcoidosis sections compared with those from tuberculosis [136]. Although the study was limited by the small number of samples analyzed (10 sarcoidosis and 12 tuberculosis), these observations indicate that M2 macrophages may exert a functional role of in the immunopathogenesis of sarcoidosis. Human CD14^+^ monocytes have the potential to differentiate into two functionally distinct subpopulations M1 and M2 macrophages. Notably, M2 macrophages, also known as “product of Th2 activation”, are the result of macrophage polarization driven by immunoregulatory cytokines such IL-4, IL-10 and TGF-β and tumor-infiltrating macrophages are typically classified as M2 and are well-known to promote malignant cells growing and immune evasion [99]. Interestingly, circulating CD14(+) monocytes from patients with sarcoidosis have altered expression of FcγR (CD16, CD32, and CD64) and complement receptors, along with increased phagocytic activity, which appear to be implicated in the high antigen load and increased circulating immune complexes observed in this disease [43].

Mycobacterial or their products can exert differential effects on immune from patients with sarcoidosis. For example, higher percentages of CD8(+) αβ (+) T-cells, with a concomitant increase in the release of pro-inflammatory cytokines was observed when peripheral blood mononuclear cells (PBMCs) from patients with sarcoidosis were stimulated in vitro with Mtb-hsp, in comparison with PBMCs from controls or from patients with tuberculosis [42]. Notably, monocytes from patients with sarcoidosis are less sensitive to apoptosis induced by mycobacterial mTB-hsp in comparison with monocytes derived from patients with tuberculosis or from healthy individuals [41]. The immunomodulatory effects of mycobacterial products have been verified in meta-analysis that included 733 participants from 13 case-control studies revealing a significantly higher immune response in sarcoidosis patients than in controls, which suggests an association to exist between mycobacteria (especially mTB) and sarcoidosis [56].

Taken together, the above discussed evidence appear to link mTB, other mycobacteria or their products with the excessive immune responses leading to sarcoidosis in a proportion of patients with sarcoidosis. Although this association in unlikely to occur in all patients and may be more significant in countries where tuberculosis is endemic.

### Potential implications of microbiota in sarcoidosis

Human microbiota composition is functionally implicated in the regulation of several physiological processes, including metabolic functions and immune homeostasis. Although gut microbiota is the most studied human microbiota niche, well-defined microbiota communities have been identified in virtually any body niche, including the skin and the respiratory tract [24, 53, 111]. In addition, the use of novel microbiome analysis techniques has shown that even internal structures such as the blood vessels, may have their own microbiota niche [51]. Alterations in the composition of this community of microorganisms has been associated with the development of several inflammatory disorders including cancer and autoimmune diseases [53, 92].

Microbiome analysis in healthy individuals reveal that airway and lung microbiome composition is similar to oropharyngeal microbiome with no evidence of site specific enrichment of bacterial communities indicating and the presence of certain microorganisms, which reach the lungs mainly via passive microaspiration, may directly modulates local immune responses [31]. Not surprisingly changes in microbe composition of the respiratory niche has been associated with inflammatory diseases [24, 140]. Since sarcoidosis manifests as a respiratory disease in more than 90% of patients, one may anticipate that the disease may be associated with specific changes in the composition of lung microbiota. Interestingly, Garzoni and colleagues analyzed the microbiome composition in BAL specimens of the lungs and respiratory tracts of individuals with sarcoidosis or with idiopathic interstitial pneumonia (IIP) and did not find any difference in the microbiome profiles of the analyzed patients [65]. It must be noted however, that although specimens were prospective analyzed, crucial limitations in this study were the small number of individuals analyzed (7 patients with sarcoidosis, 5 patients with IIP and 6 with non-IIP) and the heterogeneity of the subjects involved in the study. In addition, though microbiome composition was determined by 16S rRNA gene sequencing of BAL specimens, no extensive ultra-deep sequencing of viruses and fungi in the collection of samples was performed. In contrast, another study that included 71 patients with sarcoidosis, 15 patients with idiopathic pulmonary fibrosis and 10 healthy controls found that *Atopobium spp.* was detectable in 68% of sarcoidosis specimens but in none of healthy controls. Similarly, *Fusobacterium* spp. were also identified as sarcoidosis-associated bacteria. In the same study, gene association analysis identified an association of the variant rs2076530 in BTNL2 associated with a decrease in bacterial burden [166]. Of note, BTNL2 encodes butyrophilin-like 2, a type I transmembrane protein that negatively regulates T-cells, decreasing T-cell proliferation and cytokine release. Interestingly, the variant rs2076530 impairs the functionality of the BTNL2 protein, leading to overactivation of T-cells and consequently it has been associated with increased risk of sarcoidosis [93, 153].

Clarke and colleagues reported performed metagenomics sequencing analysis of specimens from subjects with sarcoidosis (*n* = 93) and control subjects without sarcoidosis (*n* = 72). Sarcoidosis specimens analyzed included two independent sets of formalin-fixed, paraffin-embedded (FFPE) granulomatous tissue biopsies from patients with newly identified sarcoidosis and control patients, and BAL from patients with newly diagnosed sarcoidosis and healthy control subjects. A set of 20 specimens (19 Kveim reagent and one fresh spleen) that had been prepared under sterile conditions were also analyzed. Specimens were interrogated using diverse sequencing strategies including 16S ribosomal RNA gene sequencing (for bacterial analysis), shotgun metagenomics (for virus analysis) and internal transcribed spacer ribosomal RNA gene sequencing (for fungi). In one tissue set, fungi in the Cladosporiaceae family were enriched in sarcoidosis compared with nonsarcoidosis tissues; in the other tissue set, several bacterial lineages were enrichment in sarcoidosis but not Cladosporiaceae. In BAL fluid samples, *Corynebacteria* was enriched along with a limited enrichment of Aspergillus fungi. Interestingly, in the 20 sterile-collected samples available (Kveim and spleen tissue), whole-genome shotgun sequencing analysis revealed the enrichment of several microbial lineages, especially Cladosporium [22]. Despite these promising candidates, the study did not identify any microbe signature associated with sarcoidosis, namely, no single causative microbe was consistently enriched across tissue specimen types in patients with sarcoidosis.

However, it must be noted that there were various technical limitations associated with this study. First, most samples utilized in this study were FFPE and formalin degrades DNA and also it is well-known that the de-crosslinking step necessary to undo the formalin fixation results in DNA damage which may have impaired the sensitivity for microbe detection in these samples. In addition, although in this study various negative control specimens, representing a variety of potential sources of contamination, were analyzed to exclude false positive findings, the fact that mycobacteria and *Propionibacteria* (which have been linked with sarcoidosis in previous studies) may be present in both environmental controls and patient tissues, along with the fact that mycobacteria DNA extraction is difficult, likely contributed to the false negative results reported in the study. Finally, spleen and Kveim specimens were not matched with negative control human specimens. A recent study that included 16sRNA gene sequencing of bronchoscopy obtained specimens from 31 patients with sarcoidosis and 19 with interstitial lung disease found no significant differences in the microbiome composition between the two groups, being *Firmicutes, Proteobacteria, Acinetobacter, Bacteroidetes, Fusobacteriales, and Spirochaetales* the most dominant microorganisms identified. The absence of a healthy control group and the heterogeneity of the interstitial lung disease group were important limitations in this study [13].

Furthermore, as previous studies have shown, alterations in the gut microbiome may result in the development of systemic inflammatory conditions, such as rheumatoid arthritis and atherosclerosis [75, 117, 143], which can be attributed to the pivotal role that gut microbiota play in the modulation of systemic immune response via the education of various subsets of immune cells, especially Treg cells and Th17 cells [4, 52, 106]. Is dysbiosis of the gut associated with the development or progression of sarcoidosis? To the best of our knowledge, no studies systematically assessing the gut microbiome composition in patients with sarcoidosis have been reported so far. Further studies including larger number of individuals with appropriate controls, utilizing not only freshly isolated sarcoidal specimens prepared under sterile condition, but also the gut microbiome are needed to elucidate a potential role of microbiome in the pathogenesis of this disease.

### Antimicrobials for the treatment of sarcoidosis

One may speculate that using antibiotics capable of eradicating microbes potentially linked with sarcoidosis such as *C. acnes* or Mtb could modify the clinical course of the disease. Indeed, a few studies testing the clinical efficacy of antibiotics for sarcoidosis have been reported, although most studies have been conducted in small series of cases and essentially in patients with cutaneous sarcoidosis. Measurable clinical response was observed in 10 of the 12 patients with cutaneous sarcoidosis treated with either minocycline or doxycycline, although no control group was included in this small study [7]. In a randomized, placebo-controlled single-masked trial that enrolled 30 patients with symptomatic chronic cutaneous sarcoidosis lesions, Drake et al. reported significant clinically improvements in patients receiving oral antibiotics. In this study, subjects received either a combination of levofloxacin, ethambutol, azithromycin, and rifampin (CLEAR regimen) or a placebo for 8 weeks with a 180-day follow-up [33]. The same group reported a significant improvement in forced vital capacity from baseline and better quality-of-life in 15 patients with chronic pulmonary sarcoidosis who received the CLEAR regimen for 8 weeks. The investigators also observed a normalization in the expression of tyrosine kinase Lck and NF-κB signals in the leukocytes of patients treated with the CLEAR regimen, indicating that antimicrobials reduced immune cell hyperactivation associated with the disease [34].

Although some studies utilizing electron microscopic images and various molecular biology techniques have reported the presence of infective *C. acnes* within the granulomas from some patients with sarcoidosis [47, 108], the reproducibility of those findings in a large number of patients from different populations and presenting with distinct disease phenotypes remains to be seen. In addition, it is important to consider that sarcoid granulomas constitute a chronic inflammation, rather than an acute inflammatory response and thus, even if microorganisms are the triggering factor, antibiotics could be ineffective due to the absence of an active infection within granulomas. Indeed, some studies have reported no clinical response in sarcoidosis patients treated with antibiotics [149].

Currently, a multicenter open-label clinical trial (J-ACNES) of sarcoidosis is being conducted in Japan. This study, which is the largest so far, will assess the clinical response of patients with cardiac sarcoidosis in response to antibiotic therapy for which patients will be randomized to receive either standard corticosteroid therapy plus antibiotic therapy (with known activity against *C. acnes*) or standard corticosteroid therapy alone [80]. Similarly, the utility of antibiotics is also being tested in other clinical trials of sarcoidosis in the USA and Canada (clinicaltrials.gov NCT02024555 and NCT01245036), substantiating the importance of the microbial hypothesis in the pathogenesis of sarcoidosis.

Whether or not microbes are causally implicated in the development of sarcoidosis is unclear at present. Further studies are needed in order to conclude or rule out a causal relationship between infections and sarcoidosis. In this regard, the utilization of artificial intelligence (AI) tools may be very useful for this purpose, since AI has tremendous potential in the development of algorithms for the diagnosis, treatment and prognosis of complex human diseases. AI can integrate large number of data encompassing laboratory data, imaging studies, and clinical data to better characterize disease and developing predictive models [50, 114].

## Concluding remarks and future directions

For more than one century, utilizing a variety of approaches and testing numerous specimens, researchers have tried to identify the cause of sarcoidosis to ultimately design a cure for this disease. Some of those studies have provided important insights for understanding the etiology of the sarcoidosis. For example, immunohistochemistry and molecular biology studies have delineated the cellular components of sarcoidal granulomas and the unique features that distinguish sarcoidosis from other granulomatous disorders including tuberculosis. Gene association studies have identified genetic risk factors of sarcoidosis and epidemiological studies have contributed to identify specific environmental and occupational factors strongly associated with the disease.

Several studies have tested the hypothesis that microbes may be the cause of sarcoidosis and among the numerous microorganisms investigated, mTB and *C. acnes* appear to be the strongest candidates. Although current evidence does not support a direct causal role of these pathogens in sarcoidosis, some studies have reported encouraging results on the potential utility of antibiotics for the management of patients with sarcoidosis and there is a great expectation about the results that will generate ongoing randomized clinical trials of antibiotics in sarcoidosis. Further studies are needed to better understand the specific role of the above mentioned agents in the development of sarcoidosis. For example, although articles published so far have failed to identify a lung microbiome signature in patients with sarcoidosis, data generated from those studies could not definitely rule out that an alteration in the microbiome composition of the respiratory tract or in other body niche, such as dysbiosis of the gut has some role in the development or progression of the disease. Given the complexity of the disease, future studies will require the combination of various approaches using microbiological, immunological, epidemiological and molecular biology techniques to unravel the unknown aspects of this disease. In this regard, the utilization of machine learning and deep learning algorithms will be extremely useful for future studies as artificial intelligence tools can identify causal associations that are undetectable using standard statistical analysis methods [50]. Finally, over the past years various animal models of sarcoidosis have been reported and although some of those models appear to recapitulate certain aspects of the disease [47, 77], appropriate replication of the histological and clinical features of disease is essential for the utilization of these models, not only for verifying the pathogenic role of microbe candidates but also for the identification of new therapeutic targets and surrogate biomarkers of sarcoidosis.

## Data Availability

Not applicable.

## References

[1] Adler Brandon L., Wang Catherine J., Bui Thanh-Lan, Schilperoort Hannah M., Armstrong April W. (2019). Anti-tumor necrosis factor agents in sarcoidosis: A systematic review of efficacy and safety. Seminars in Arthritis and Rheumatism.

[2] Al-Harbi A, Al-Otaibi S, Abdulrahman A, Al-Jahdali F, Al-Harbi F, Bamefleh H, Gamdi M, Al-Jahdali H (2017). Lung granuloma: a clinicopathologic study of 158 cases. Ann Thorac Med.

[3] Arkema EV, Cozier YC (2018). Epidemiology of sarcoidosis: current findings and future directions. Ther Adv Chronic Dis.

[4] Arpaia N, Campbell C, Fan X, Dikiy S, Van Der Veeken J, Deroos P, Liu H, Cross JR, Pfeffer K, Coffer PJ, Rudensky AY (2013). Metabolites produced by commensal bacteria promote peripheral regulatory T-cell generation. Nature.

[5] Asakawa N, Uchida K, Sakakibara M, Omote K, Noguchi K, Tokuda Y, Kamiya K, Hatanaka KC, Matsuno Y, Yamada S, Asakawa K, Fukasawa Y, Nagai T, Anzai T, Ikeda Y, Ishibashi-Ueda H, Hirota M, Orii M, Akasaka T, Uto K, Shingu Y, Matsui Y, Morimoto SI, Tsutsui H, Eishi Y (2017). Immunohistochemical identification of Propionibacterium acnes in granuloma and inflammatory cells of myocardial tissues obtained from cardiac sarcoidosis patients. PLoS One.

[6] Babu K (2013). Sarcoidosis in tuberculosis-endemic regions: India. J Ophthalmic Inflamm Infect.

[7] Bachelez H, Senet P, Cadranel J, Kaoukhov A, Dubertret L (2001). The use of tetracyclines for the treatment of sarcoidosis. Arch Dermatol.

[8] Badar F, Azfar SF, Ahmad I, Yasmeen S, Kirmani S (2011). Diagnostic difficulties in differentiating sarcoidosis from tuberculosis. Oman Med J.

[9] Badgwell C, Rosen T (2007). Cutaneous sarcoidosis therapy updated. J Am Acad Dermatol.

[10] Baughman RP, Grutters JC (2015). New treatment strategies for pulmonary sarcoidosis: antimetabolites, biological drugs, and other treatment approaches. Lancet Respir Med.

[11] Baughman RP, Lower EE (2007). Evidence-based therapy for cutaneous sarcoidosis. Clin Dermatol.

[12] Baughman RP, Lower EE (2015). Treatment of sarcoidosis. Clin Rev Allergy Immunol.

[13] Becker A, Vella G, Galata V, Rentz K, Beisswenger C, Herr C, Walter J, Tierling S, Slevogt H, Keller A, Bals R (2019). The composition of the pulmonary microbiota in sarcoidosis - an observational study. Respir Res.

[14] Bickett AN, Lower EE, Baughman RP (2018). Sarcoidosis diagnostic score: a systematic evaluation to enhance the diagnosis of sarcoidosis. Chest.

[15] Bonifazi M, Gasparini S, Alfieri V, Renzoni EA (2017). Pulmonary Sarcoidosis. Semin Respir Crit Care Med.

[16] Bowe C, Jenssen F, Espinoza A (2017). Case report of sarcoidosis as a great mimicker in various populations. J La State Med Soc.

[17] Braun NA, Celada LJ, Herazo-Maya JD, Abraham S, Shaginurova G, Sevin CM, Grutters J, Culver DA, Dworski R, Sheller J, Massion PP, Polosukhin VV, Johnson JE, Kaminski N, Wilkes DS, Oswald-Richter KA, Drake WP (2014). Blockade of the programmed death-1 pathway restores sarcoidosis CD4(+) T-cell proliferative capacity. Am J Respir Crit Care Med.

[18] Broos CE, Van Nimwegen M, Kleinjan A, Ten Berge B, Muskens F, In 'T Veen JC, Annema JT, Lambrecht BN, Hoogsteden HC, Hendriks RW, Kool M, Van Den Blink B (2015). Impaired survival of regulatory T cells in pulmonary sarcoidosis. Respir Res.

[19] Brownell I, Ramírez-Valle F, Sanchez M, Prystowsky S (2011). Evidence for mycobacteria in sarcoidosis. Am J Respir Cell Mol Biol.

[20] Casanova N, Zhou T, Knox KS, Garcia JGN (2015). Identifying novel biomarkers in sarcoidosis using genome-based approaches. Clin Chest Med.

[21] Chapelon-Abric Catherine, Saadoun David, Marie Isabelle, Comarmond Cloé, Desbois Anne Claire, Domont Fanny, Savey Léa, Cacoub Patrice (2017). Sarcoidosis with Takayasu arteritis: a model of overlapping granulomatosis. A report of seven cases and literature review. International Journal of Rheumatic Diseases.

[22] Clarke EL, Lauder AP, Hofstaedter CE, Hwang Y, Fitzgerald AS, Imai I, Biernat W, Rękawiecki B, Majewska H, Dubaniewicz A, Litzky LA, Feldman MD, Bittinger K, Rossman MD, Patterson KC, Bushman FD, Collman RG (2018). Microbial lineages in sarcoidosis. A metagenomic analysis tailored for low-microbial content samples. Am J Respir Crit Care Med.

[23] Coquart N, Cadelis G, Tressières B, Cordel N (2015). Epidemiology of sarcoidosis in afro-Caribbean people: a 7-year retrospective study in Guadeloupe. Int J Dermatol.

[24] Costa André Nathan, Costa Felipe Marques da, Campos Silvia Vidal, Salles Roberta Karla, Athanazio Rodrigo Abensur (2018). The pulmonary microbiome: challenges of a new paradigm. Jornal Brasileiro de Pneumologia.

[25] Crouser ED, Fingerlin TE, Yang IV, Maier LA, Nana-Sinkam P, Collman RG, Kaminski N (2017). Application of “omics” and systems biology to sarcoidosis research. Ann Am Thorac Soc.

[26] Cummings MM, Hammarsten JF (1962). Sarcoidosis. Annu Rev Med.

[27] Cunningham-Bussel A, Zhang T, Nathan CF (2013). Nitrite produced by mycobacterium tuberculosis in human macrophages in physiologic oxygen impacts bacterial ATP consumption and gene expression. Proc Natl Acad Sci U S A.

[28] Dahan A, Brines M, Niesters M, Cerami A, Van Velzen M (2016). Targeting the innate repair receptor to treat neuropathy. Pain Rep.

[29] Darlington P, Haugom-Olsen H, Von Sivers K, Wahlström J, Runold M, Svjatoha V, Porwit A, Eklund A, Grunewald J (2012). T-cell phenotypes in bronchoalveolar lavage fluid, blood and lymph nodes in pulmonary sarcoidosis--indication for an airborne antigen as the triggering factor in sarcoidosis. J Intern Med.

[30] Derler AM, Eisendle K, Baltaci M, Obermoser G, Zelger B (2009). High prevalence of ‘Borrelia-like’ organisms in skin biopsies of sarcoidosis patients from Western Austria. J Cutan Pathol.

[31] Dickson RP, Erb-Downward JR, Freeman CM, Mccloskey L, Falkowski NR, Huffnagle GB, Curtis JL. Bacterial topography of the healthy human Lower respiratory tract. MBio. 2017;8(1). 10.1128/mBio.02287-16. 10.1128/mBio.02287-16 PMC5312084 28196961

[32] Dimitriou F, Frauchiger AL, Urosevic-Maiwald M, Naegeli MC, Goldinger SM, Barysch M, Franzen D, Kamarachev J, Braun R, Dummer R, Mangana J (2018). Sarcoid-like reactions in patients receiving modern melanoma treatment. Melanoma Res.

[33] Drake WP, Oswald-Richter K, Richmond BW, Isom J, Burke VE, Algood H, Braun N, Taylor T, Pandit KV, Aboud C, Yu C, Kaminski N, Boyd AS, King LE (2013). Oral antimycobacterial therapy in chronic cutaneous sarcoidosis: a randomized, single-masked, placebo-controlled study. JAMA Dermatol.

[34] Drake WP, Richmond BW, Oswald-Richter K, Yu C, Isom JM, Worrell JA, Shipley GR (2013). Effects of broad-spectrum antimycobacterial therapy on chronic pulmonary sarcoidosis. Sarcoidosis Vasc Diffuse Lung Dis.

[35] Du Bois RM, Goh N, Mcgrath D, Cullinan P (2003). Is there a role for microorganisms in the pathogenesis of sarcoidosis?. J Intern Med.

[36] Dubaniewicz A (2010). Mycobacterium tuberculosis heat shock proteins and autoimmunity in sarcoidosis. Autoimmun Rev.

[37] Dubaniewicz A (2013). Microbial and human heat shock proteins as ‘danger signals’ in sarcoidosis. Hum Immunol.

[38] Dubaniewicz A, Dubaniewicz-Wybieralska M, Sternau A, Zwolska Z, Izycka-Swieszewska E, Augustynowicz-Kopec E, Skokowski J, Singh M, Zimnoch L (2006). Mycobacterium tuberculosis complex and mycobacterial heat shock proteins in lymph node tissue from patients with pulmonary sarcoidosis. J Clin Microbiol.

[39] Dubaniewicz A, Holownia A, Kalinowski L, Wybieralska M, Dobrucki IT, Singh M (2013). Is mycobacterial heat shock protein 16 kDa, a marker of the dormant stage of mycobacterium tuberculosis, a sarcoid antigen?. Hum Immunol.

[40] Dubaniewicz A, Kämpfer S, Singh M (2006). Serum anti-mycobacterial heat shock proteins antibodies in sarcoidosis and tuberculosis. Tuberculosis (Edinb).

[41] Dubaniewicz A, Trzonkowski P, Dubaniewicz-Wybieralska M, Singh M, Myśliwski A (2006). Comparative analysis of mycobacterial heat shock proteins-induced apoptosis of peripheral blood mononuclear cells in sarcoidosis and tuberculosis. J Clin Immunol.

[42] Dubaniewicz A, Trzonkowski P, Dubaniewicz-Wybieralska M, Singh M, Myśliwski A (2007). Mycobacterial heat shock protein-induced blood T lymphocytes subsets and cytokine pattern: comparison of sarcoidosis with tuberculosis and healthy controls. Respirology.

[43] Dubaniewicz A, Typiak M, Wybieralska M, Szadurska M, Nowakowski S, Staniewicz-Panasik A, Rogoza K, Sternau A, Deeg P, Trzonkowski P (2012). Changed phagocytic activity and pattern of Fcγ and complement receptors on blood monocytes in sarcoidosis. Hum Immunol.

[44] Dubrey S, Shah S, Hardman T, Sharma R (2014). Sarcoidosis: the links between epidemiology and aetiology. Postgrad Med J.

[45] Duchemann Boris, Annesi-Maesano Isabella, Jacobe de Naurois Camille, Sanyal Shreosi, Brillet Pierre-Yves, Brauner Michel, Kambouchner Marianne, Huynh Sophie, Naccache Jean Marc, Borie Raphael, Piquet Jacques, Mekinian Arsène, Virally Jerôme, Uzunhan Yurdagul, Cadranel Jacques, Crestani Bruno, Fain Olivier, Lhote Francois, Dhote Robin, Saidenberg-Kermanac'h Nathalie, Rosental Paul-André, Valeyre Dominique, Nunes Hilario (2017). Prevalence and incidence of interstitial lung diseases in a multi-ethnic county of Greater Paris. European Respiratory Journal.

[46] Eishi Y (2013). Etiologic aspect of sarcoidosis as an allergic endogenous infection caused by Propionibacterium acnes. Biomed Res Int.

[47] Eishi Y (2013). Etiologic link between sarcoidosis and Propionibacterium acnes. Respir Investig.

[48] Eishi Y, Suga M, Ishige I, Kobayashi D, Yamada T, Takemura T, Takizawa T, Koike M, Kudoh S, Costabel U, Guzman J, Rizzato G, Gambacorta M, Du Bois R, Nicholson AG, Sharma OP, Ando M (2002). Quantitative analysis of mycobacterial and propionibacterial DNA in lymph nodes of Japanese and European patients with sarcoidosis. J Clin Microbiol.

[49] Eklund A (2003). Aetiology, pathogenesis and treatment of sarcoidosis. J Intern Med.

[50] Espinoza J. Luis (2018). Machine learning for tackling microbiota data and infection complications in immunocompromised patients with cancer. Journal of Internal Medicine.

[51] Espinoza J., Ai Suzue, Matsumura Itaru (2018). New Insights on the Pathogenesis of Takayasu Arteritis: Revisiting the Microbial Theory. Pathogens.

[52] Espinoza JL, Elbadry MI, Nakao S (2016). An altered gut microbiota may trigger autoimmune-mediated acquired bone marrow failure syndromes. Clin Immunol.

[53] Espinoza JL, Minami M (2018). Sensing bacterial-induced DNA damaging effects. Front Immunol.

[54] Esteves T, Aparicio G, Garcia-Patos V (2016). Is there any association between sarcoidosis and infectious agents?: a systematic review and meta-analysis. BMC Pulm Med.

[55] Fakhri G, Akel R, Salem Z, Tawil A, Tfayli A (2017). Pulmonary sarcoidosis activation following neoadjuvant Pembrolizumab plus chemotherapy combination therapy in a patient with non-small cell lung cancer: a case report. Case Rep Oncol.

[56] Fang C, Huang H, Xu Z (2016). Immunological evidence for the role of mycobacteria in sarcoidosis: a meta-analysis. PLoS One.

[57] Fares A (2013). Factors influencing the seasonal patterns of infectious diseases. Int J Prev Med.

[58] Farrokhi S, Abbasirad N, Movahed A, Khazaei HA, Pishjoo M, Rezaei N (2017). TLR9-based immunotherapy for the treatment of allergic diseases. Immunotherapy.

[59] Feng Y, Zhou J, Gu C, Ding Y, Wan H, Ni L, Niu W (2013). Association of six well-characterized polymorphisms in TNF-α and TNF-β genes with sarcoidosis: a meta-analysis. PLoS One.

[60] Fingerlin TE, Hamzeh N, Maier LA (2015). Genetics of sarcoidosis. Clin Chest Med.

[61] Fischer A, Ellinghaus D, Nutsua M, Hofmann S, Montgomery CG, Iannuzzi MC, Rybicki BA, Petrek M, Mrazek F, Pabst S, Grohé C, Grunewald J, Ronninger M, Eklund A, Padyukov L, Mihailovic-Vucinic V, Jovanovic D, Sterclova M, Homolka J, Nöthen MM, Herms S, Gieger C, Strauch K, Winkelmann J, Boehm BO, Brand S, Büning C, Schürmann M, Ellinghaus E, Baurecht H, Lieb W, Nebel A, Müller-Quernheim J, Franke A, Schreiber S, Consortium G (2015). Identification of immune-relevant factors conferring sarcoidosis genetic risk. Am J Respir Crit Care Med.

[62] Flores RJ, Flaherty KR, Jin Z, Bokhari S. The prognostic value of quantitating and localizing F-18 FDG uptake in cardiac sarcoidosis. J Nucl Cardiol. 2018. 10.1007/s12350-018-01504-y. 10.1007/s12350-018-01504-y 30421379

[63] Fussner Lynn A., Karlstedt Erin, Hodge David O., Fine Nowell M., Kalra Sanjay, Carmona Eva M., Utz James P., Isaac Debra L., Cooper Leslie T. (2018). Management and outcomes of cardiac sarcoidosis: a 20‐year experience in two tertiary care centres. European Journal of Heart Failure.

[64] Gabrilovich MI, Walrath J, Van Lunteren J, Nethery D, Seifu M, Kern JA, Harding CV, Tuscano L, Lee H, Williams SD, Mackay W, Tomashefski JF, Silver RF (2013). Disordered toll-like receptor 2 responses in the pathogenesis of pulmonary sarcoidosis. Clin Exp Immunol.

[65] Garzoni C, Brugger SD, Qi W, Wasmer S, Cusini A, Dumont P, Gorgievski-Hrisoho M, Mühlemann K, Von Garnier C, Hilty M (2013). Microbial communities in the respiratory tract of patients with interstitial lung disease. Thorax.

[66] Georas SN, Chapman TJ, Crouser ED (2016). Sarcoidosis and T-helper cells. Th1, Th17, or Th17.1?. Am J Respir Crit Care Med.

[67] Gerke AK, Judson MA, Cozier YC, Culver DA, Koth LL (2017). Disease burden and variability in sarcoidosis. Ann Am Thorac Soc.

[68] Ghossein RA, Ross DG, Salomon RN, Rabson AR (1994). A search for mycobacterial DNA in sarcoidosis using the polymerase chain reaction. Am J Clin Pathol.

[69] Gkiozos I, Kopitopoulou A, Kalkanis A, Vamvakaris IN, Judson MA, Syrigos KN (2018). Sarcoidosis-like reactions induced by checkpoint inhibitors. J Thorac Oncol.

[70] Goto H, Usui Y, Umazume A, Uchida K, Eishi Y (2017). As a possible pathogen of granuloma in patients with ocular sarcoidosis. Br J Ophthalmol.

[71] Grunewald J, Eklund A (2007). Sex-specific manifestations of Löfgren’s syndrome. Am J Respir Crit Care Med.

[72] Gupta D, Agarwal R, Aggarwal AN, Jindal SK (2007). Molecular evidence for the role of mycobacteria in sarcoidosis: a meta-analysis. Eur Respir J.

[73] Hattori T, Konno S, Shijubo N, Yamaguchi T, Sugiyama Y, Honma S, Inase N, Ito YM, Nishimura M (2018). Nationwide survey on the organ-specific prevalence and its interaction with sarcoidosis in Japan. Sci Rep.

[74] Heinle R, Chang C (2014). Diagnostic criteria for sarcoidosis. Autoimmun Rev.

[75] Horta-Baas G, Romero-Figueroa MDS, Montiel-Jarquín AJ, Pizano-Zárate ML, García-Mena J, Ramírez-Durán N (2017). Intestinal Dysbiosis and rheumatoid arthritis: a link between gut microbiota and the pathogenesis of rheumatoid arthritis. J Immunol Res.

[76] Hosoda Y, Chiba Y (1964). The relationship of sarcoidosis to tuberculosis. Acta Med Scand Suppl.

[77] Hu Y, Yibrehu B, Zabini D, Kuebler WM (2017). Animal models of sarcoidosis. Cell Tissue Res.

[78] Huang H, Lu Z, Jiang C, Liu J, Wang Y, Xu Z (2013). Imbalance between Th17 and regulatory T-cells in sarcoidosis. Int J Mol Sci.

[79] Ibitoye RT, Wilkins A, Scolding NJ (2017). Neurosarcoidosis: a clinical approach to diagnosis and management. J Neurol.

[80] Ishibashi K, Eishi Y, Tahara N, Asakura M, Sakamoto N, Nakamura K, Takaya Y, Nakamura T, Yazaki Y, Yamaguchi T, Asakura K, Anzai T, Noguchi T, Yasuda S, Terasaki F, Hamasaki T, Kusano K (2018). Japanese antibacterial drug Management for Cardiac Sarcoidosis (J-ACNES): a multicenter, open-label, randomized, controlled study. J Arrhythm.

[81] Ishihara M, Ishida T, Isogai E, Kimura K, Oritsu M, Matsui Y, Isogai H, Ohno S (1996). Detection of antibodies to Borrelia species among patients with confirmed sarcoidosis in a region where Lyme disease is nonendemic. Graefes Arch Clin Exp Ophthalmol.

[82] Ishihara M, Ohno S, Ono H, Isogai E, Kimura K, Isogai H, Aoki K, Ishida T, Suzuki K, Kotake S, Hiraga Y (1998). Seroprevalence of anti-Borrelia antibodies among patients with confirmed sarcoidosis in a region of Japan where Lyme borreliosis is endemic. Graefes Arch Clin Exp Ophthalmol.

[83] James WE, Baughman R (2018). Treatment of sarcoidosis: grading the evidence. Expert Rev Clin Pharmacol.

[84] Judson MA (2018). The diagnosis of sarcoidosis: attempting to apply rigor to arbitrary and circular reasoning. Chest.

[85] Kachamakova-Trojanowska N, Jazwa-Kusior A, Szade K, Kasper L, Soja J, Andrychiewicz A, Jakiela B, Plutecka H, Sanak M, Jozkowicz A, Sladek K, Dulak J (2018). Molecular profiling of regulatory T cells in pulmonary sarcoidosis. J Autoimmun.

[86] Kandolin R, Lehtonen J, Kupari M (2016). Cardiac sarcoidosis. J Intern Med.

[87] Karakaya B, Kaiser Y, Van Moorsel CHM, Grunewald J (2017). Löfgren’s syndrome: diagnosis, management, and disease pathogenesis. Semin Respir Crit Care Med.

[88] Khosravi A, Yáñez A, Price JG, Chow A, Merad M, Goodridge HS, Mazmanian SK (2014). Gut microbiota promote hematopoiesis to control bacterial infection. Cell Host Microbe.

[89] Kim CH, Park J, Kim M (2014). Gut microbiota-derived short-chain fatty acids, T cells, and inflammation. Immune Netw.

[90] Kishore A, Petrek M (2018). Next-generation sequencing based HLA typing: deciphering Immunogenetic aspects of sarcoidosis. Front Genet.

[91] Korsten P, Tampe B, Konig MF, Nikiphorou E (2018). Sarcoidosis and autoimmune diseases: differences, similarities and overlaps. Curr Opin Pulm Med.

[92] Kriss M, Hazleton KZ, Nusbacher NM, Martin CG, Lozupone CA (2018). Low diversity gut microbiota dysbiosis: drivers, functional implications and recovery. Curr Opin Microbiol.

[93] Li Y, Wollnik B, Pabst S, Lennarz M, Rohmann E, Gillissen A, Vetter H, Grohé C (2006). BTNL2 gene variant and sarcoidosis. Thorax.

[94] Litinsky I, Elkayam O, Flusser G, Segal R, Yaron M, Caspi D (2002). Sarcoidosis: TB or not TB?. Ann Rheum Dis.

[95] Liu Y, Qiu L, Wang Y, Aimurola H, Zhao Y, Li S, Xu Z (2016). The circulating Treg/Th17 cell ratio is correlated with relapse and treatment response in pulmonary sarcoidosis patients after corticosteroid withdrawal. PLoS One.

[96] London J, Grados A, Fermé C, Charmillon A, Maurier F, Deau B, Crickx E, Brice P, Chapelon-Abric C, Haioun C, Burroni B, Alifano M, Le Jeunne C, Guillevin L, Costedoat-Chalumeau N, Schleinitz N, Mouthon L, Terrier B (2014). Sarcoidosis occurring after lymphoma: report of 14 patients and review of the literature. Medicine (Baltimore).

[97] Marshall TG, Marshall FE (2004). Sarcoidosis succumbs to antibiotics--implications for autoimmune disease. Autoimmun Rev.

[98] Martens H, Zöllner B, Zissel G, Burdon D, Schlaak M, Müller-Quernheim J (1997). Anti-Borrelia burgdorferi immunoglobulin seroprevalence in pulmonary sarcoidosis: a negative report. Eur Respir J.

[99] Martinez FO, Gordon S (2014). The M1 and M2 paradigm of macrophage activation: time for reassessment. F1000Prime Rep.

[100] Mañá J, Rubio-Rivas M, Villalba N, Marcoval J, Iriarte A, Molina-Molina M, Llatjos R, García O, Martínez-Yélamos S, Vicens-Zygmunt V, Gámez C, Pujol R, Corbella X (2017). Multidisciplinary approach and long-term follow-up in a series of 640 consecutive patients with sarcoidosis: cohort study of a 40-year clinical experience at a tertiary referral center in Barcelona, Spain. Medicine (Baltimore).

[101] Mian A, Ray A (2018). Thoracic sarcoidosis versus tuberculosis: need for a multi-disciplinary approach. Indian J Radiol Imaging.

[102] Mirsaeidi M, Machado RF, Schraufnagel D, Sweiss NJ, Baughman RP (2015). Racial difference in sarcoidosis mortality in the United States. Chest.

[103] Miyazaki K, Yamada H, Tamura T, Shiozawa T, Satoh H (2018). Pulmonary tuberculosis developed in sarcoidosis patients. Tuberk Toraks.

[104] Moller DR, Rybicki BA, Hamzeh NY, Montgomery CG, Chen ES, Drake W, Fontenot AP (2017). Genetic, immunologic, and environmental basis of sarcoidosis. Ann Am Thorac Soc.

[105] Mortaz E, Masjedi MR, Abedini A, Matroodi S, Kiani A, Soroush D, Adcock IM (2016). Common features of tuberculosis and sarcoidosis. Int J Mycobacteriol.

[106] Nagano Y, Itoh K, Honda K (2012). The induction of Treg cells by gut-indigenous clostridium. Curr Opin Immunol.

[107] Nagata K, Eishi Y, Uchida K, Yoneda K, Hatanaka H, Yasuhara T, Nagata M, Sotozono C, Kinoshita S (2017). Immunohistochemical detection of Propionibacterium acnes in the retinal granulomas in patients with ocular sarcoidosis. Sci Rep.

[108] Negi M, Takemura T, Guzman J, Uchida K, Furukawa A, Suzuki Y, Iida T, Ishige I, Minami J, Yamada T, Kawachi H, Costabel U, Eishi Y (2012). Localization of propionibacterium acnes in granulomas supports a possible etiologic link between sarcoidosis and the bacterium. Mod Pathol.

[109] Nessrine A, Zahra AF, Taoufik H (2014). Musculoskeletal involvement in sarcoidosis. J Bras Pneumol.

[110] Newman KL, Newman LS (2012). Occupational causes of sarcoidosis. Curr Opin Allergy Clin Immunol.

[111] O'dwyer DN, Dickson RP, Moore BB (2016). The lung microbiome, immunity, and the pathogenesis of chronic lung disease. J Immunol.

[112] Osaki M, Adachi H, Gomyo Y, Yoshida H, Ito H (1997). Detection of mycobacterial DNA in formalin-fixed, paraffin-embedded tissue specimens by duplex polymerase chain reaction: application to histopathologic diagnosis. Mod Pathol.

[113] Oswald-Richter KA, Beachboard DC, Seeley EH, Abraham S, Shepherd BE, Jenkins CA, Culver DA, Caprioli RM, Drake WP (2012). Dual analysis for mycobacteria and propionibacteria in sarcoidosis BAL. J Clin Immunol.

[114] Owais Muhammad, Arsalan Muhammad, Choi Jiho, Park Kang Ryoung (2019). Effective Diagnosis and Treatment through Content-Based Medical Image Retrieval (CBMIR) by Using Artificial Intelligence. Journal of Clinical Medicine.

[115] Pasadhika S, Rosenbaum JT (2015). Ocular Sarcoidosis. Clin Chest Med.

[116] Perng RP, Chou KT, Chu H, Chung YM (2007). Familial sarcoidosis in Taiwan. J Formos Med Assoc.

[117] Picchianti-Diamanti A, Rosado MM, D’amelio R (2017). Infectious agents and inflammation: the role of microbiota in autoimmune arthritis. Front Microbiol.

[118] Post J, Hull P (2012). Tattoo reactions as a sign of sarcoidosis. CMAJ.

[119] Reich JM (2016). Epidemiology of sarcoidosis. Mayo Clin Proc.

[120] Riley EA (1950). Boeck’s sarcoid; a review based upon a clinical study of fifty-two cases. Am Rev Tuberc.

[121] Rivera NV, Ronninger M, Shchetynsky K, Franke A, Nöthen MM, Müller-Quernheim J, Schreiber S, Adrianto I, Karakaya B, Van Moorsel CH, Navratilova Z, Kolek V, Rybicki BA, Iannuzzi MC, Petrek M, Grutters JC, Montgomery C, Fischer A, Eklund A, Padyukov L, Grunewald J (2016). High-density genetic mapping identifies new susceptibility variants in sarcoidosis phenotypes and shows genomic-driven phenotypic differences. Am J Respir Crit Care Med.

[122] Rosen Y (2007). Pathology of sarcoidosis. Semin Respir Crit Care Med.

[123] Rossides Marios, Grunewald Johan, Eklund Anders, Kullberg Susanna, Di Giuseppe Daniela, Askling Johan, Arkema Elizabeth V. (2018). Familial aggregation and heritability of sarcoidosis: a Swedish nested case−control study. European Respiratory Journal.

[124] Saboor SA, Johnson NM, Mcfadden J (1992). Detection of mycobacterial DNA in sarcoidosis and tuberculosis with polymerase chain reaction. Lancet.

[125] Saidha S, Sotirchos ES, Eckstein C (2012). Etiology of sarcoidosis: does infection play a role?. Yale J Biol Med.

[126] Saketkoo LA, Baughman RP (2016). Biologic therapies in the treatment of sarcoidosis. Expert Rev Clin Immunol.

[127] Sakthivel P, Bruder D (2017). Mechanism of granuloma formation in sarcoidosis. Curr Opin Hematol.

[128] Sakthivel P, Grunewald J, Eklund A, Bruder D, Wahlström J (2016). Pulmonary sarcoidosis is associated with high-level inducible co-stimulator (ICOS) expression on lung regulatory T cells--possible implications for the ICOS/ICOS-ligand axis in disease course and resolution. Clin Exp Immunol.

[129] Sato H, Nagai S, Du Bois RM, Handa T, Suginoshita Y, Ohta K, Welsh KI, Izumi T (2007). HLA-DQB1 0602 allele is associated with splenomegaly in Japanese sarcoidosis. J Intern Med.

[130] Sato H, Woodhead FA, Ahmad T, Grutters JC, Spagnolo P, Van Den Bosch JM, Maier LA, Newman LS, Nagai S, Izumi T, Wells AU, Du Bois RM, Welsh KI (2010). Sarcoidosis HLA class II genotyping distinguishes differences of clinical phenotype across ethnic groups. Hum Mol Genet.

[131] Sawahata M, Sugiyama Y (2016). An epidemiological perspective of the pathology and etiology of sarcoidosis. Sarcoidosis Vasc Diffuse Lung Dis.

[132] Sayah DM, Bradfield JS, Moriarty JM, Belperio JA, Lynch JP (2017). Cardiac involvement in sarcoidosis: evolving concepts in diagnosis and treatment. Semin Respir Crit Care Med.

[133] Scadding JG (1960). Mycobacterium tuberculosis in the aetiology of sarcoidosis. Br Med J.

[134] Schnerch J, Prasse A, Vlachakis D, Schuchardt KL, Pechkovsky DV, Goldmann T, Gaede KI, Müller-Quernheim J, Zissel G (2016). Functional toll-like receptor 9 expression and CXCR3 ligand release in pulmonary sarcoidosis. Am J Respir Cell Mol Biol.

[135] Schupp Jonas Christian, Freitag-Wolf Sandra, Bargagli Elena, Mihailović-Vučinić Violeta, Rottoli Paola, Grubanovic Aleksandar, Müller Annegret, Jochens Arne, Tittmann Lukas, Schnerch Jasmin, Olivieri Carmela, Fischer Annegret, Jovanovic Dragana, Filipovic Snežana, Videnovic-Ivanovic Jelica, Bresser Paul, Jonkers René, O'Reilly Kate, Ho Ling-Pei, Gaede Karoline I., Zabel Peter, Dubaniewicz Anna, Marshall Ben, Kieszko Robert, Milanowski Janusz, Günther Andreas, Weihrich Anette, Petrek Martin, Kolek Vitezslav, Keane Michael P., O'Beirne Sarah, Donnelly Seamas, Haraldsdottir Sigridur Olina, Jorundsdottir Kristin B., Costabel Ulrich, Bonella Francesco, Wallaert Benoît, Grah Christian, Peroš-Golubičić Tatjana, Luisetti Mauritio, Kadija Zamir, Pabst Stefan, Grohé Christian, Strausz János, Vašáková Martina, Sterclova Martina, Millar Ann, Homolka Jiří, Slováková Alena, Kendrick Yvonne, Crawshaw Anjali, Wuyts Wim, Spencer Lisa, Pfeifer Michael, Valeyre Dominique, Poletti Venerino, Wirtz Hubertus, Prasse Antje, Schreiber Stefan, Krawczak Michael, Müller-Quernheim Joachim (2018). Phenotypes of organ involvement in sarcoidosis. European Respiratory Journal.

[136] Shamaei M, Mortaz E, Pourabdollah M, Garssen J, Tabarsi P, Velayati A, Adcock IM (2018). Evidence for M2 macrophages in granulomas from pulmonary sarcoidosis: a new aspect of macrophage heterogeneity. Hum Immunol.

[137] Shariatmaghani Somayeh, Salari Roshanak, Sahebari Maryam, Tabrizi Payman Shalchian, Salari Masoumeh (2019). Musculoskeletal Manifestations of Sarcoidosis: A Review Article. Current Rheumatology Reviews.

[138] Song GG, Kim JH, Lee YH (2014). Associations between TNF-α −308 a/G and lymphotoxin-α +252 a/G polymorphisms and susceptibility to sarcoidosis: a meta-analysis. Mol Biol Rep.

[139] Spagnolo P, Sato H, Grunewald J, Brynedal B, Hillert J, Mañá J, Wells AU, Eklund A, Welsh KI, Du Bois RM (2008). A common haplotype of the C-C chemokine receptor 2 gene and HLA-DRB1*0301 are independent genetic risk factors for Löfgren's syndrome. J Intern Med.

[140] Sulaiman Imran, Wu Benjamin G., Li Yonghua, Scott Adrienne S., Malecha Patrick, Scaglione Benjamin, Wang Jing, Basavaraj Ashwin, Chung Samuel, Bantis Katrina, Carpenito Joseph, Clemente Jose C., Shen Nan, Bessich Jamie, Rafeq Samaan, Michaud Gaetene, Donington Jessica, Naidoo Charissa, Theron Grant, Schattner Gail, Garofano Suzette, Condos Rany, Kamelhar David, Addrizzo-Harris Doreen, Segal Leopoldo N. (2018). Evaluation of the airway microbiome in nontuberculous mycobacteria disease. European Respiratory Journal.

[141] Suzuki Y, Uchida K, Takemura T, Sekine M, Tamura T, Furukawa A, Hebisawa A, Sakakibara Y, Awano N, Amano T, Kobayashi D, Negi M, Kakegawa T, Wada Y, Ito T, Suzuki T, Akashi T, Eishi Y (2018). Propionibacterium acnes-derived insoluble immune complexes in sinus macrophages of lymph nodes affected by sarcoidosis. PLoS One.

[142] Taflin C, Miyara M, Nochy D, Valeyre D, Naccache JM, Altare F, Salek-Peyron P, Badoual C, Bruneval P, Haroche J, Mathian A, Amoura Z, Hill G, Gorochov G (2009). FoxP3+ regulatory T cells suppress early stages of granuloma formation but have little impact on sarcoidosis lesions. Am J Pathol.

[143] Tang WH, Kitai T, Hazen SL (2017). Gut microbiota in cardiovascular health and disease. Circ Res.

[144] Ten Berge B, Paats MS, Bergen IM, Van Den Blink B, Hoogsteden HC, Lambrecht BN, Hendriks RW, Kleinjan A (2012). Increased IL-17A expression in granulomas and in circulating memory T cells in sarcoidosis. Rheumatology (Oxford).

[145] Testi I, Tognon MS, Gupta V. Diagnostic challenges in granulomatous uveitis: tuberculosis or sarcoidosis? Ocul Immunol Inflamm. 2018:1–3. 10.1080/09273948.2018.1491997. 10.1080/09273948.2018.1491997 29993306

[146] Tetzlaff MT, Nelson KC, Diab A, Staerkel GA, Nagarajan P, Torres-Cabala CA, Chasen BA, Wargo JA, Prieto VG, Amaria RN, Curry JL (2018). Granulomatous/sarcoid-like lesions associated with checkpoint inhibitors: a marker of therapy response in a subset of melanoma patients. J Immunother Cancer.

[147] Thulasidoss K, Asokan L, Chandra P, Rejliwal P. The clinical conundrum of diagnosing and treating systemic sarcoidosis in a high TB burden area. BMJ Case Rep. 2017;2017. 10.1136/bcr-2016-218741. 10.1136/bcr-2016-218741 PMC5612530 28500120

[148] Timmermans WM, Van Laar JA, Van Hagen PM, Van Zelm MC (2016). Immunopathogenesis of granulomas in chronic autoinflammatory diseases. Clin Transl Immunology.

[149] Toutous-Trellu L, Ninet B, Rohner P, Auckenthaler R, Saurat JH, Pechère M (2000). Three cases of cutaneous sarcoidosis: search for bacterial agent by the 16S RNA gene analysis and treatment with antibiotics. Dermatology.

[150] Trompette A, Gollwitzer ES, Yadava K, Sichelstiel AK, Sprenger N, Ngom-Bru C, Blanchard C, Junt T, Nicod LP, Harris NL, Marsland BJ (2014). Gut microbiota metabolism of dietary fiber influences allergic airway disease and hematopoiesis. Nat Med.

[151] Tøndell A, Moen T, Børset M, Salvesen Ø, Rø AD, Sue-Chu M (2014). Bronchoalveolar lavage fluid IFN-γ+ Th17 cells and regulatory T cells in pulmonary sarcoidosis. Mediat Inflamm.

[152] Ungprasert P, Crowson CS, Matteson EL (2016). Seasonal variation in incidence of sarcoidosis: a population-based study, 1976-2013. Thorax.

[153] Valentonyte R, Hampe J, Huse K, Rosenstiel P, Albrecht M, Stenzel A, Nagy M, Gaede KI, Franke A, Haesler R, Koch A, Lengauer T, Seegert D, Reiling N, Ehlers S, Schwinger E, Platzer M, Krawczak M, Müller-Quernheim J, Schürmann M, Schreiber S (2005). Sarcoidosis is associated with a truncating splice site mutation in BTNL2. Nat Genet.

[154] Van Moorsel CH, Christiani DC (2012). Genetic susceptibility to sarcoidosis, a chronic inflammatory disorder. Am J Respir Crit Care Med.

[155] Van Velzen M, Heij L, Niesters M, Cerami A, Dunne A, Dahan A, Brines M (2014). ARA 290 for treatment of small fiber neuropathy in sarcoidosis. Expert Opin Investig Drugs.

[156] Vogel WV, Guislain A, Kvistborg P, Schumacher TN, Haanen JB, Blank CU (2012). Ipilimumab-induced sarcoidosis in a patient with metastatic melanoma undergoing complete remission. J Clin Oncol.

[157] Webber MP, Yip J, Zeig-Owens R, Moir W, Ungprasert P, Crowson CS, Hall CB, Jaber N, Weiden MD, Matteson EL, Prezant DJ (2017). Post-9/11 sarcoidosis in WTC-exposed firefighters and emergency medical service workers. Respir Med.

[158] Wolin A, Lahtela EL, Anttila V, Petrek M, Grunewald J, Van Moorsel CHM, Eklund A, Grutters JC, Kolek V, Mrazek F, Kishore A, Padyukov L, Pietinalho A, Ronninger M, Seppänen M, Selroos O, Lokki ML (2017). SNP variants in major histocompatibility complex are associated with sarcoidosis susceptibility-a joint analysis in four European populations. Front Immunol.

[159] Wu CH, Chung PI, Wu CY, Chen YT, Chiu YW, Chang YT, Liu HN (2017). Comorbid autoimmune diseases in patients with sarcoidosis: a nationwide case-control study in Taiwan. J Dermatol.

[160] Yang G, Eishi Y, Raza A, Rojas H, Achiriloaie A, De Los Reyes K, Raghavan R (2018). Propionibacterium acnes-associated neurosarcoidosis: a case report with review of the literature. Neuropathology.

[161] Yatsynovich Y, Dittoe N, Petrov M, Maroz N (2018). Cardiac sarcoidosis: a review of contemporary challenges in diagnosis and treatment. Am J Med Sci.

[162] Zella S, Kneiphof J, Haghikia A, Gold R, Woitalla D, Thöne J (2018). Successful therapy with rituximab in three patients with probable neurosarcoidosis. Ther Adv Neurol Disord.

[163] Zhou Y, Wei YR, Zhang Y, Du SS, Baughman RP, Li HP (2015). Real-time quantitative reverse transcription-polymerase chain reaction to detect propionibacterial ribosomal RNA in the lymph nodes of Chinese patients with sarcoidosis. Clin Exp Immunol.

[164] Ziegenhagen MW, Müller-Quernheim J (2003). The cytokine network in sarcoidosis and its clinical relevance. J Intern Med.

[165] Ziemer M, Grabner T, Eisendle K, Baltaci M, Zelger B (2008). Granuloma annulare--a manifestation of infection with Borrelia?. J Cutan Pathol.

[166] Zimmermann Alexandra, Knecht Henrik, Häsler Robert, Zissel Gernot, Gaede Karoline I., Hofmann Sylvia, Nebel Almut, Müller-Quernheim Joachim, Schreiber Stefan, Fischer Annegret (2017). Atopobium and Fusobacterium as novel candidates for sarcoidosis-associated microbiota. European Respiratory Journal.

